# Transcriptomic Analysis and the Expression of Disease-Resistant Genes in *Oryza meyeriana* under Native Condition

**DOI:** 10.1371/journal.pone.0144518

**Published:** 2015-12-07

**Authors:** Bin He, Xiang Tao, Yinghong Gu, Changhe Wei, Xiaojie Cheng, Suqin Xiao, Zaiquan Cheng, Yizheng Zhang

**Affiliations:** 1 Key Laboratory of Bio-resources and Eco-environment, Ministry of Education, Sichuan Key Laboratory of Molecular Biology and Biotechnology, College of Life Sciences, Sichuan University, Chengdu, 610064, China; 2 Biotechnology & Genetic Resources Institute, Yunnan Academy of Agricultural Sciences, Kunming, 650223, China; Michigan State University, UNITED STATES

## Abstract

*Oryza meyeriana* (*O*. *meyeriana*), with a GG genome type (2n = 24), accumulated plentiful excellent characteristics with respect to resistance to many diseases such as rice shade and blast, even immunity to bacterial blight. It is very important to know if the diseases-resistant genes exist and express in this wild rice under native conditions. However, limited genomic or transcriptomic data of *O*. *meyeriana* are currently available. In this study, we present the first comprehensive characterization of the *O*. *meyeriana* transcriptome using RNA-seq and obtained 185,323 contigs with an average length of 1,692 bp and an N50 of 2,391 bp. Through differential expression analysis, it was found that there were most tissue-specifically expressed genes in roots, and next to stems and leaves. By similarity search against protein databases, 146,450 had at least a significant alignment to existed gene models. Comparison with the *Oryza sativa* (*japonica*-type Nipponbare and *indica*-type 93–11) genomes revealed that 13% of the *O*. *meyeriana* contigs had not been detected in *O*. *sativa*. Many diseases-resistant genes, such as bacterial blight resistant, blast resistant, rust resistant, fusarium resistant, cyst nematode resistant and downy mildew gene, were mined from the transcriptomic database. There are two kinds of rice bacterial blight-resistant genes (*Xa1* and *Xa26*) differentially or specifically expressed in *O*. *meyeriana*. The 4 *Xa1* contigs were all only expressed in root, while three of *Xa26* contigs have the highest expression level in leaves, two of *Xa26* contigs have the highest expression profile in stems and one of *Xa26* contigs was expressed dominantly in roots. The transcriptomic database of *O*. *meyeriana* has been constructed and many diseases-resistant genes were found to express under native condition, which provides a foundation for future discovery of a number of novel genes and provides a basis for studying the molecular mechanisms associated with disease resistance in *O*. *meyeriana*.

## Introduction

The world is facing a new challenge with global population by the middle of last century[1]. Increasing food production to feed the world’s population is an even greater challenge considering that agriculture is experiencing greater competition for land, water and energy, as well as, the effects of substantial climate change and the unintended effects of crop production on the environment. Part of the solution to increase food production on the same or less cultivated land lies in exploiting the subset of genes lost during the domestication process and subsequent targeted breeding. Currently, these valuable genes are found only in the progenitor species gene pool for crop cultivars. Cultivated plants having desirable genes were utilized in intensive breeding projects which focused on increasing yield for particular environments and management systems but this process has narrowed the genetic diversity[2]. For cultivated plants, this unexploited genetic material includes both landraces and the more exotic wild relatives. Improving our understanding of this tertiary gene pool and exploiting it for crop improvement are paramount to meeting the challenges of feeding the world in this century through the integration of classical genetics and genomics-enabled research paradigms.

The *Oryza* genus includes two cultivated species, Asian rice, *O*. *sativa*, and African rice, *O*. *glaberrima*. The 22 wild species composing the *Oryza* genus are characterized by eleven different genomes identified as the A-, B-, C-, D-, E-, F-, G-, H-, J-, K-and L-genomes and arranged in the following 10 genome types AA, BB, CC, BBCC, CCDD, EE, FF, GG, HHJJ and KKLL[3, 4]. Across long-term survival competition and natural selection under environments with some extreme conditions, wild rice accumulated so plentiful excellent characteristics and turned out to be the excellent gene pool and irreplaceable material basis for improving the cultivated varieties[5, 6]. *Oryza meyeriana* (2n = 24, GG), broadly distributed throughout Southeast Asia, is a perennial species with characteristics of shade tolerance, resistance to blast and immunity to bacterial blight (BB). As early as 1991, hybrids were produced from a cross between *O*. *sativa* and *O*. *meyeriana* to breed rice resistant varieties[7]. Subsequently, the line Y73 was selected for a high level of bacterial blight resistance from the hybrid progeny of an asymmetric somatic hybridization between an *O*. *meyeriana* and an *O*. *sativa* subsp. *japonica* cultivar[8]. In spite of its overall inferior appearance, *O*. *meyeriana* has furnished genes for developing disease resistance varieties[9, 10]. Meanwhile, *O*. *meyeriana*, GG genomes, has diverged from *Oryza* species in an earlier era and is considered to retain lots of primordial behaviors so that it is deemed to a desirable objective for studying the phylogenetic relationships of *Oryza* species[11]. To make better use of this potential, more genomic information is required, but there are only few batches of mRNAs or full-length cDNAs (FLcDNAs) of *O*. *meyeriana* in public databases, and no genome sequence is available.

Sequencing and analysis of expressed sequence tags (ESTs) has become a primary strategy for functional genomic studies in plants, especially for non-model organisms, including novel gene discovery, gene expression profiling, molecular marker development, and accurate genome annotation[12]. After completing the full genome sequence of *O*. *sativa ssp*. *japonica* cv. Nipponbare and the draft genome sequence of the *O*. *sativa ssp*. *indica* cv. 9311 through a map-based sequencing strategy and through a whole genome shotgun sequencing approach, respectively, much efforts were involved into rice ESTs projects[13, 14]. To date, approximately 1,279,471 ESTs and more than 60,000 FLcDNA sequences of cultivated rice are currently available in public databases[15]. However, the genomic studies of rice wild relatives are still in their infancy with the exception of the generation of 5,211 leaf ESTs from the *O*. *minuta* (BBCC genome), 1,888 leaf FLcDNAs from the *O*. *rufipogon* (AA genome) and 71,367 root unique sequences from the *O*. *longistaminata* (AA genome)[15–17]. Further more, genes that might be expressed in different organs are underrepresented in EST studies.

Therefore, a comprehensive survey of ESTs in *O*. *meyeriana* was undertaken to provide an overview of *O*. *meyeriana* transcriptome and thus a molecular basis for the identification of useful genes. As newly developed massively parallel Illumina sequencing allows rapid generation of sequence data and deep sequencing coverage with reducing labor and cost[12, 18], we here characterized the first global transcriptome of the wild rice species *O*. *meyeriana* by Illumina sequencing technology. This led to the discovery of a huge amount of novel genes which will facilitate gene mining, especially those diseases-resistant genes expressing under the native conditions, and provide a basis for comparative studies within the genus *Oryza*.

## Results

### Sequencing and *de novo* assembly of *O*. *meyeriana* transcriptome

**Fig 1:**
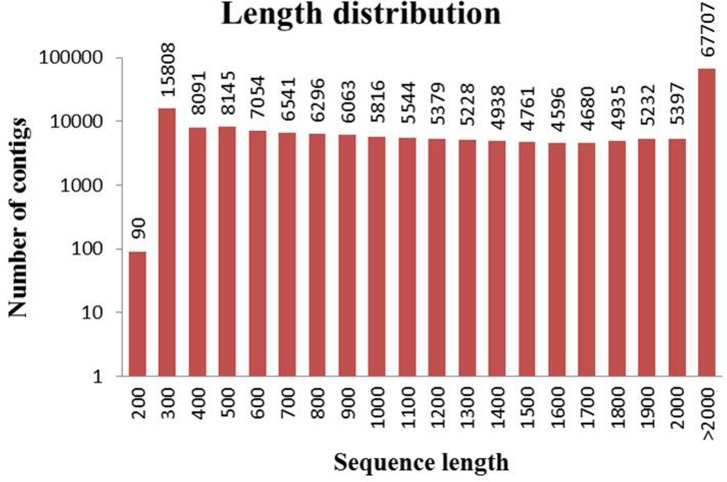
Length distributions of *O*. *meyeriana* transcriptome.

To obtain an integrated transcriptome of *O*. *meyeriana*, samples collected from roots, stems and leaves were used for RNA-Seq analysis. There were 55,285,080, 52,810,076 and 54,037,340 pair-end (PE) of 90 bp reads obtained from roots, leaves and stems samples respectively. Through comparison, we choose the Single-Assembler Multiple-Parameters of Oases as assembly strategy on the basis of our previous study[19]. Finally we obtained 185,323 contigs with an average length of 1,692 bp and an N50 of 2,391 bp (Table 1). Almost all of the contigs were longer than 200 bp and 118,397 contigs (64%) were longer than 1,000 bp. The size distribution of these contigs is shown in Fig 1.

**Table 1:** Summary of *de novo* sequence assembly of *O*. *meyeriana*.

Transcriptome evaluation	Length (bp)
Total length	313,584,308
Number of contig	185,323
Maximum contig length	21,120
Average contig length	1,692
N50 length	2,391

An optimality criterion for a novel *de novo* assembled transcriptome is how well it recapitulates previously determined sequences for the target species, and how well it represents sequences from related organisms. Through sequence homology search with 44 and 523 identified genes in *O*. *meyeriana* and *O*. *sativa*, we evaluated the gene coverage of assembled transcriptome. The results showed that all 44 complete protein-coding genes from *O*. *meyeriana* were present in our assembled transcriptome. 428 of 523 (81.84%) proteins from *O*. *sativa* could hit our assembled transcriptome (details shown in S1 Table).

Since most transcripts derived from polyadenylated RNA are expected to code for proteins, the optimal assembly results could produce long and complete ORFs (open reading frames) as many as possible. From the results of predicted ORFs, 133,901 ORFs were predicted from our assembled transcriptome with 87,760 (66%) ORFs longer than 899bp. Among them, there were 84,128 intact ORFs, accounting for 63% of all predicted ORFs.

When all reads were mapped to the contigs, there were 95.48% of the reads aligned back to the contigs, which demonstrated that almost all reads were utilized for the *de novo* assembly. In addition, the accuracy of the assembly results was up to 88%.

On the basis of the results of above evaluation, the quality of the assembled transcriptome is good enough for functional annotation and further analysis.

### Functional annotation and characterization of *O*. *meyeriana* transcripts

Several complementary methods were used to annotate the assembled sequences. First, the consensus sequences were annotated for sequence similarities using the BLASTX translated sequence comparison against the NCBI non-redundant (NR) protein database. Among the 185,323 assembled contigs, 146,450 (79.02%) had at least a significant alignment to existing gene models in the NR database at an E-value cut-off of 1e-5.

**Fig 2:**
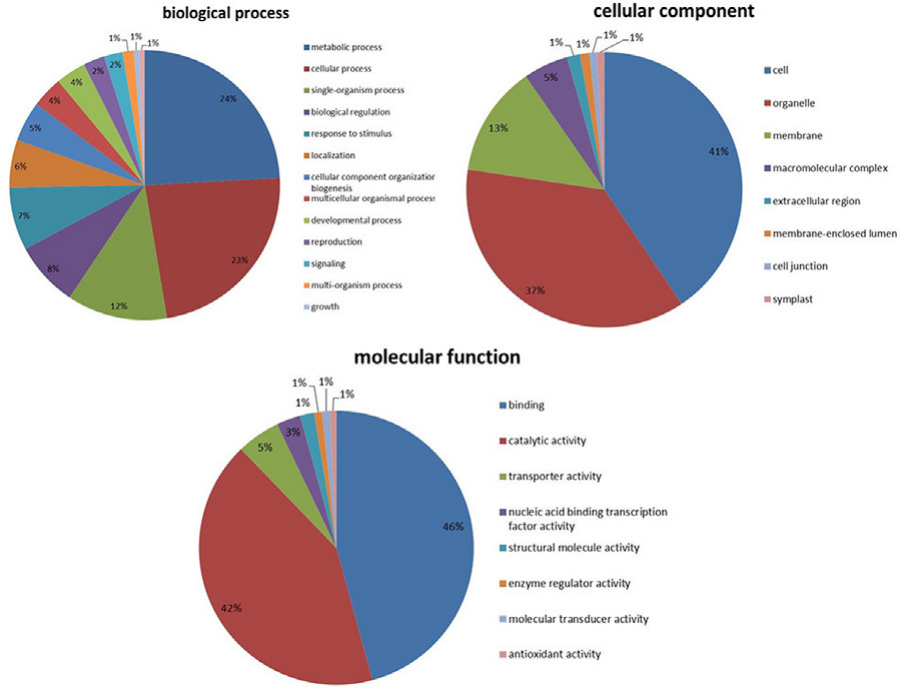
Gene Ontology classification of all *O*. *meyeriana* transcripts.

Second, sequences that had matches in NR databases achieved GO (Gene Ontology) annotations with the Uniprot database. Of these, 126,448 were assigned to one or more GO terms, for a total of 611,020 term occurrences, which were assigned in cellular component, molecular function and biological process. Among the various cellular component, ignoring unknown and other cellular component categories, cell (41%), organelle (37%) and membrane (13%) were most highly represented. The genes involved in other important cellular components such as extracellular region, membrane-enclosed lumen, macromolecular complex, cell junction and symplast, were also identified through GO annotations. Similarly, binding (46%) and catalytic activity (42%) were most represented among the various molecular functions, and metabolic process (24%) and cellular process (23%) were most represented in the biological process categories (Fig 2).

### KEGG pathways analysis

To identify biological pathways of *O*. *meyeriana*, the assembled contigs were annotated with Enzyme Commission (EC) numbers from BLASTX alignments against the KEGG database (E-value ≤ 1e-5). The assigned EC numbers were subsequently mapped to the reference pathways. As a result, a total of 8,720 assembled sequences were found to be involved in 300 predicted KEGG metabolic pathways. Purine metabolism (1,021), starch and source metabolism (673) and pyrimidine metabolism pathways (525) enriched the greatest number of sequences. The top 20 pathways with the greatest number of sequences are shown in Table 2. These results provide a valuable resource for investigating metabolic pathways in *O*. *meyeriana*.

**Table 2:** The top 20 pathways with the highest contig numbers.

Num	Pathway	All genes with pathway annotation (8720)	Pathway ID
**1**	Purine metabolism	1021 (11.71%)	ko00230
**2**	starch and sucrose metabolism	673 (7.72%)	ko00500
**3**	pyrimidine metabolism	525 (6.02%)	ko00240
**4**	Thiamine metabolism	378 (4.34%)	ko00730
**5**	Amino sugar and nucleotide sugar metabolism	238 (2.73%)	ko00520
**6**	Pyruvate metabolism	215 (2.47%)	ko00620
**7**	Target of Rapamycin signaling pathway	199 (2.28%)	ko04150
**8**	Glycolysis / Gluconeogenesis	191 (2.19%)	ko00010
**9**	T cell receptor signaling pathway	179 (2.05%)	ko04660
**10**	Glycerolipid metabolism	172 (1.97%)	ko00561
**11**	Glycerophospholipid metabolism	159 (1.82%)	ko00564
**12**	Pentose and glucuronate interconversions	156 (1.79%)	ko00040
**13**	Cysteine and methionine metabolism	151 (1.73%)	ko00270
**14**	Glyoxylate and dicarboxylate metabolism	141 (1.62%)	ko00630
**15**	Porphyrin and chlorophyll metabolism	130 (1.49%)	ko00860
**16**	Glutathione metabolism	126 (1.45%)	ko00480
**17**	Valine, leucine and isoleucine degradation	119 (1.37%)	ko00280
**18**	Drug metabolism—other enzymes	114 (1.31%)	ko00983
**19**	Arginine and proline metabolism	114 (1.31%)	ko00330
**20**	Phenylalanine metabolism	112 (1.29%)	ko00360

### Composition analysis of *O*. *meyeriana* transcripts

GC content analysis showed that the average GC content of *O*. *meyeriana* transcripts is up to 51.74%, and average GC contents of the first, second and third base in *O*. *meyeriana* transcripts are 57.25%, 44.33% and 53.63%. Compared with *Arabidopsis* (dicot reference) and rice (monocot reference), the average GC content of *O*. *meyeriana* transcripts (51.74%) was lower than that of rice (55%) and much higher than that of *Arabidopsis* (42.5%) [20].

According to the results of codon usage, we noted that the most frequently used stop codon is TAA (occurs in 43.5% of all transcripts) followed by TGA (31.5%) and TAG (25%). TGA is the most frequently used stop codon in *Arabidopsis* (44%) and rice (43%), but in *Arabidopsis* TAA (36%) is more frequent than TAG (20%), whereas similar frequencies of TAG (30%) and TAA (27%) are used in rice[21]. In addition, the code usage of leucine showed the strongest preference of CTC (23%), the most frequently used codon, is 2.9 times that TTA (8%). The detailed codon preference was shown in S2 Table.

The transcript/EST-based markers are important resource for determining functional genetic variation. Among the various molecular markers, SSRs (Simple Sequence Repeats) are highly polymorphic, easier to develop and serve as rich resource of diversity. For identification of SSRs, all the *O*. *meyeriana* transcripts were searched with perl script MISA (Microsatellite identification tool). We identified a total of 60,498 SSRs in 43,128 (23.27%) transcripts of *O*. *meyeriana* with frequency of one SSR per 5.18 kb of the sequence (Table 3). The Tri-nucleotide SSRs represented the largest fraction (74.02%) of SSRs followed by di-nucleotide (23.30%) SSRs. In addition, a small fraction of tetra-(1.82%), penta-(0.56%) and hexa-nucleotide (0.31%) SSRs were identified in *O*. *meyeriana* transcripts.

**Table 3:** Statistics of SSRs identified in *O*. *meyeriana* transcriptome.

**SSR mining**	
Total number of sequences examined	185323
Total size of examined sequences (bp)	313,584,308
Total number of identified SSRs	60498
Number of SSR containing sequences	43128
Number of sequences containing more than 1 SSR	11920
Number of SSRs present in compound formation	6249
Frequency of SSRs	One per 5.18 kb
**Distribution of SSRs in different repeat types**	
Dinucleotide	14093 (23.30%)
Trinucleotide	44778 (74.02%)
Tetranucleotide	1102 (1.82%)
Pentanucleotide	338 (0.56%)
Hexanucleotide	187 (0.31%)
Total	60498 (100%)

### Differentially and specifically expressed genes among three tissues

Gene expression was calculated in accordance with the method of FPKM (fragments per kilobase per million). On the basis of the applied criteria [FDR (False Discovery Rate) ≤0.0001 and log_2_fold-change (log_2_FC)≥1], 114,932 contigs (62% of all contigs) were identified as significant differential expression genes (DEGs) between leaves and root tissues, which comprised 52,816 up-regulated contigs (accounting for 46% of all significant differentially expressed genes) and 62,116 down-regulated contigs (accounting for 54%) in *O*. *meyeriana* transcripts. Between leaves and stem tissues, 31,505 DEGs (17% of all contigs) were identified, including 17,232 up-regulated contigs (55%) and 14,273 down-regulated contigs (45%). There were 117,487 contigs (63% of all contigs) identified as significant DEGs between root and stem tissues, which comprised 65,950 up-regulated genes (56%) and 51,537 down-regulated genes (44%).

**Fig 3:**
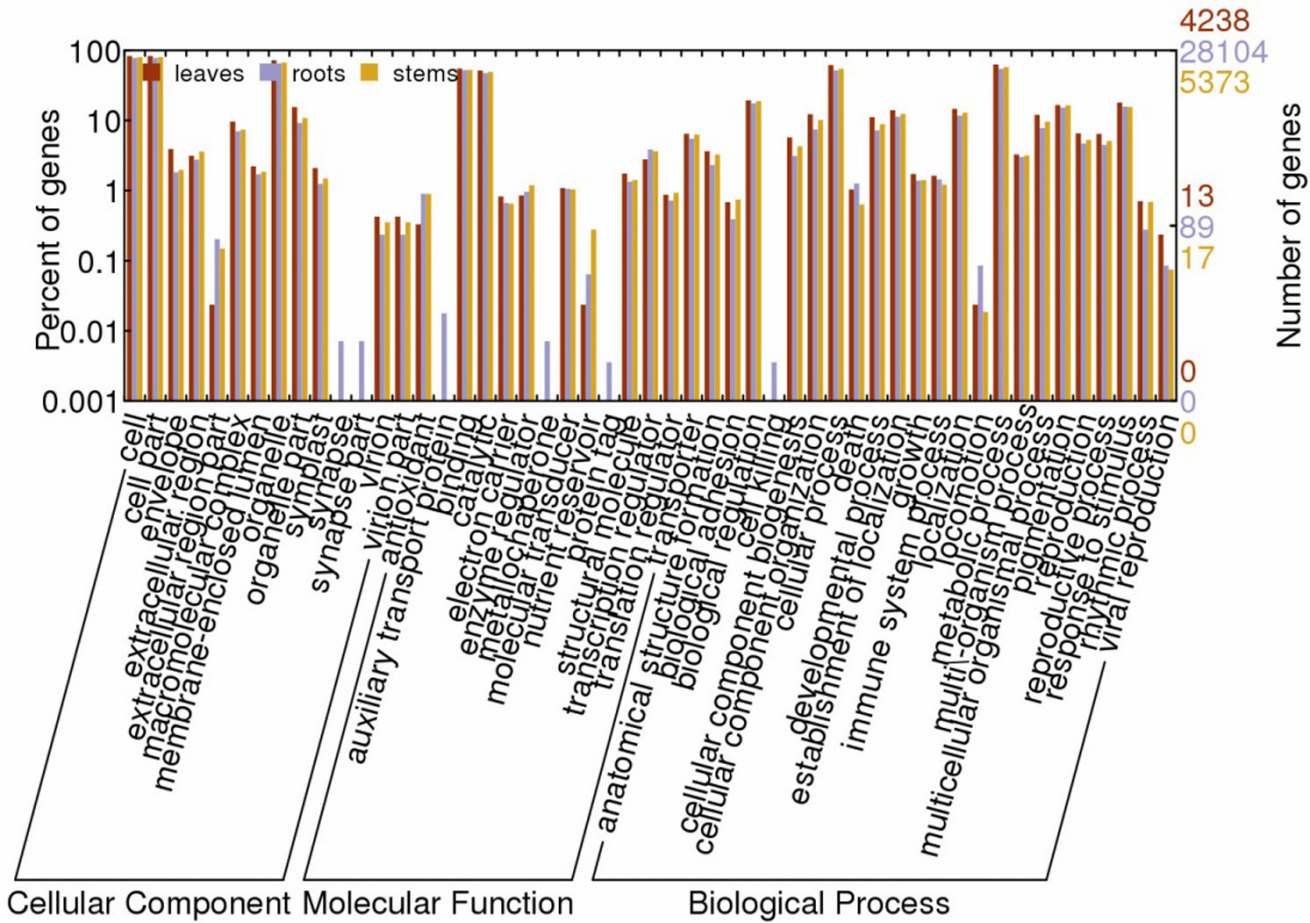
Gene Ontology classification of tissue-specifically expressed genes.

In addition, we identified tissue-specifically expressed genes (TSEGs), in which there were the most TSEGs in roots (45,942), and next to stems (9,051) and leaves (7,250). To determine their functions, all TSEGs were mapped to terms in the GO database. Of these TSEGs, there were 28,104, 5,373 and 4,238 contigs assigned to one or more GO terms in root, stem and leaves, respectively. The results from the comparison of GO annotations showed significant difference, though all of them could be categorized into three main categories: biological process, cellular component and molecular function. In each of the three main categories, ‘synapse’ and ‘synapse part’ terms enriched in the ‘cellular component’ category, ‘auxiliary transport protein’, ‘metallochaperone’ and ‘protein tag’ term enriched in the ‘molecular function’ category, ‘cell killing’ and ‘locomotion’ term enriched in the ‘biological process’ category revealed dominant behavior in root tissue (Fig 3). Meanwhile, the term ‘nutrient reservoir’ enriched in the ‘molecular function’ and the term ‘extracellular region part’ enriched in the ‘cellular component’ revealed dominant behavior in stem and leave tissue, respectively.

### Comparison of *O*. *meyeriana* transcriptome with the *O*. *sativa* genome

To investigate the phylogenetic relationship between *O*. *meyeriana* and *O*. *sativa*, all of the reads from *O*. *meyeriana* were aligned to genomic sequences of two *O*. *sativa* varieties, *japonica*-type Nipponbare and *indica*-type 93–11. 57.65% and 57.09% of reads could only be aligned to *indica* and *japonica* genome, respectively.

When the contigs of *O*. *meyeriana* were compared with genomic sequences of two *O*. *sativa* varieties, it was found that total 165,646 (89.38%) *O*. *meyeriana* contigs had similarity hits. Among these contigs, 160,453 could be aligned to the genome sequences of both *indica* and *japonica*. In addition, 161,467 (87.13%) and 162,262 (87.56%) contigs were anchored in *japonica* and *indica* genome, respectively. These contigs could be mapped on all the 12 rice chromosomes of Nipponbare and 93–11 with almost equal distribution and the chromosome 1, 2, 3 harbored large number of contigs accounting for approximately 39% of a total of 63,703 and 64,175 contigs (Table 4). The distribution corresponds well to the size of the chromosomes. Nevertheless, the comparative analysis on the alignment of reads and contigs with *Japonica* and *Indica* genomes suggested that the differentiation between *indica* and *japonica* has not occurred in *O*. *meyeriana* to some extent.

**Table 4:** Distribution of the contigs mapped to genomes of two rice cultivars.

Chromosome^a^	*japonica*		*indica*	
	NO. of contigs	Percentage	NO. of contigs	Percentage
01 (43.26 Mb)	22821	14.1	22800	14.1
02 (35.93 Mb)	19101	11.8	19198	11.8
03 (36.41 Mb)	21781	13.5	22177	13.7
04 (35.28 Mb)	13655	8.5	13832	8.5
05 (29.89 Mb)	13616	8.4	13687	8.4
06 (31.25 Mb)	13397	8.3	13465	8.3
07 (29.70 Mb)	12196	7.6	12025	7.4
08 (28.44 Mb)	10637	6.6	10527	6.5
09 (23.01 Mb)	8464	5.2	8532	5.3
10 (23.13 Mb)	8308	5.1	8075	5.0
11 (28.51 Mb)	7415	4.6	7141	4.4
12 (27.50 Mb)	8328	5.2	8155	5.0
Unkonown^b^	1748	1.1	2648	1.6
Total	161467	100	162262	100

^a^ The length of 12 Nipponbare (*japonica* cv.) chromosomes is indicated

^b^ Genes not assembled into chromosomes

**Fig 4:**
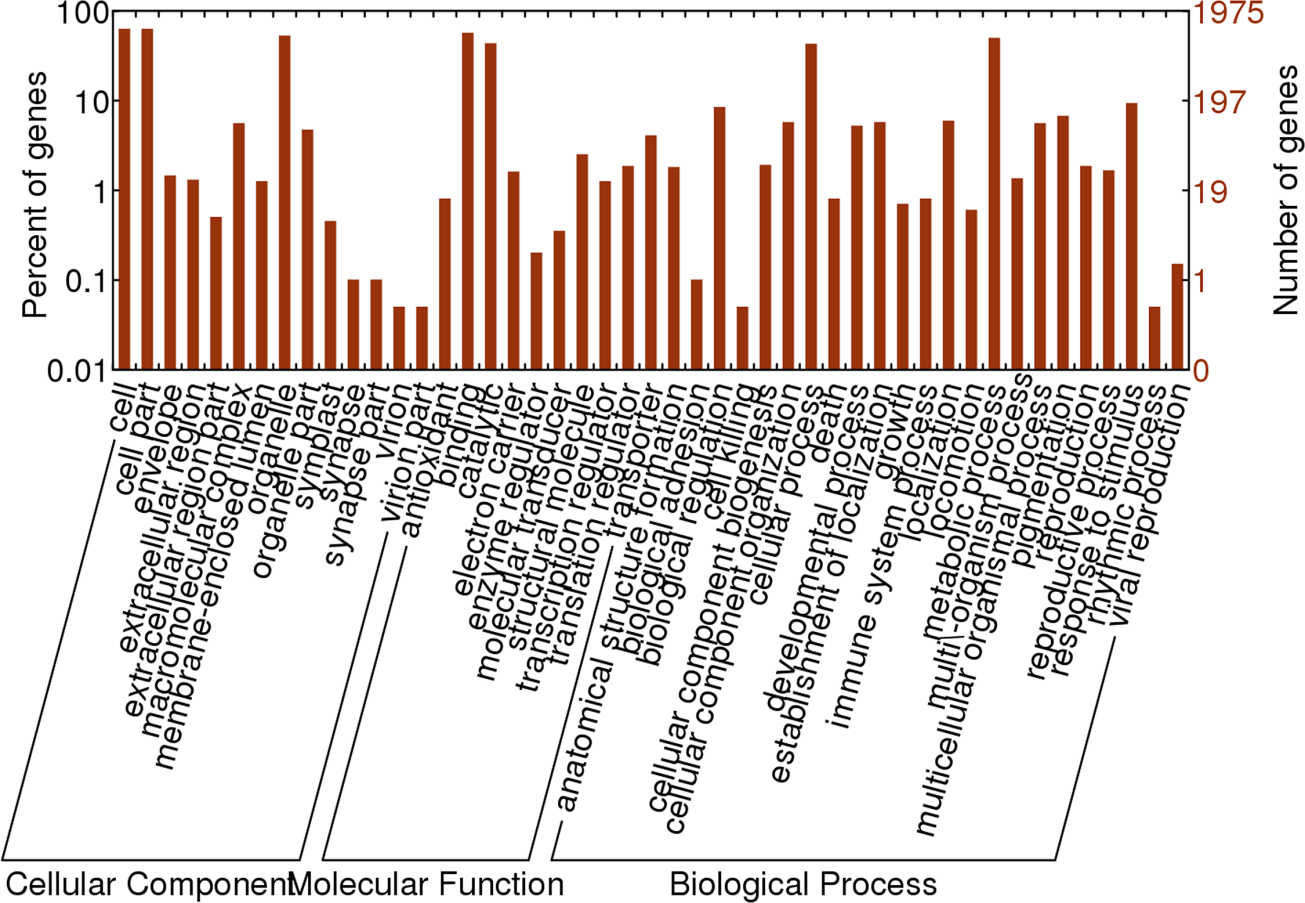
Gene Ontology classification of *O*. *meyeriana*-specifically expressed genes.

Among the remained 19,677 contigs (10.62% of all contigs), only a very small number (3,572, 1.93%) had a significant hit in NCBI NR protein database. In addition, based on the similarity search above, we conducted a GO analysis for functional classification to these contigs of *O*. *meyeriana*. The results demonstrated that there were only 1,975 contigs assigned to one or more GO terms. The contigs grouped in cell and cell part were highly represented. For molecular functions, ‘binding’ was the most prevalent GO term, followed by ‘catalytic’. For cellular components, the most represented category was ‘cells’ and ‘cell part’, followed by and ‘organelle’. For biological processes, ‘metabolic processes’ and ‘cellular processes’ showed high percentage (Fig 4). In addition, we investigated the functional domains of these genes in which no hits with the *O*. *sativa* genomes was detected. A total of 1466 functional domains were identified in 1466 genes, including some domains related to disease resistance, such as NB-ARC (a nucleotide-binding adaptor shared by APAF-1, certain R gene products and CED-4) domain, LRR-TM (leucine rich repeat-transmembrane) domain and so on.

### Candidate genes involved in the plant-pathogen interaction pathway

Due to the characters of resistance to blast and immunization to bacterial blight of *O*. *meyeriana*, we searched for candidate genes involved in the plant-pathogen interaction pathway. There were 13 contigs annotated as encoding enzymes involved in plant-pathogen interaction based on the KEGG pathway assignments. Plants lack animal-like adaptive immunity mechanisms, but have evolved a specific system with multiple layers against invading pathogens.

The primary response includes the perception of pathogens by cell-surface pattern-recognition receptors (PRRs) and is referred to as PAMP-triggered immunity (PTI). Activation of the flagellin receptor FLS2 and EFR (Elongation factor-Tu receptor) triggers Mitogen-Activated Protein Kinase (MAPK) signaling pathway that activates defense genes for antimicrobial compounds. We identified one gene (Contig_13135) homologous to FLS2 with identity of 99% and one gene (Contig_82291) homologous to EF-Tu with identity of 100%. The increase in the cytosolic Ca^2+^ concentration is also a regulator for production of reactive oxygen species and localized programmed cell death/hypersensitive response. Cyclic nucleotide gated channel (CNGC), calcium-dependent protein kinase (CDPK), calcium-binding protein CML (CaMCML), respiratory burst oxidase (Rboh) and nitric-oxide synthase (NOS) are necessary during this process. We identified three of these enzymes, except for Rboh and NOS, in the pathway (Contig_31872 annotated as CNGC, Contig_76733 annotated as CDPK and Contig_128397 annotated as CaMCML).

**Fig 5:**
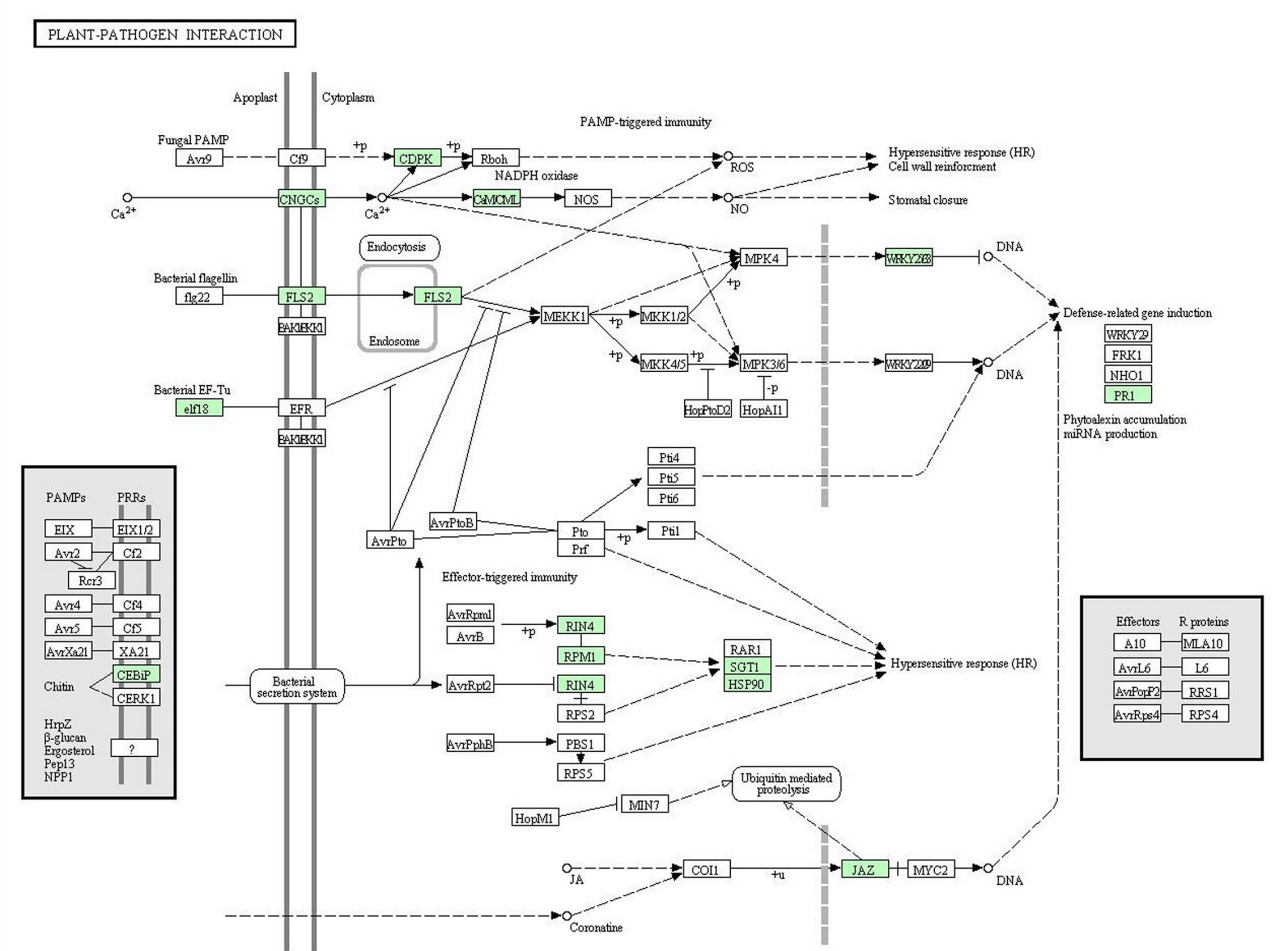
Contigs predicted to be involved in the plant-pathogen interaction pathway in *O*. *meyeriana*. Blue indicates contigs expressed in *O*. *meyeriana*.

The secondary response is called effector-triggered immunity (ETI). Pathogens can acquire the ability to suppress PTI by directly injecting effector proteins into the plant cell through secretion systems. In addition, pathogens can manipulate plant hormone signaling pathways to evade host immune responses using coronatine toxin. Some plants possess specific intracellular surveillance proteins (R proteins) to monitor the presence of pathogen virulence proteins. This ETI occurs with localized programmed cell death to arrest pathogen growth, resulting in cultivar-specific disease resistance (Fig 5). When referring to the plant hormone signaling pathways, we identified one gene (Contig_90332) homologous to jasmonate ZIM domain-containing protein (JAZ) in *O*. *meyeriana*. On the other hand, four R proteins, RPM1-interacting protein 4 (RIN4), disease resistance protein RPM1 (RPM1), suppressor of G2 allele of SKP1 (SGT1) and heat shock protein (HSP90), were also identified in *O*. *meyeriana* (Contig_123815 annotated as RIN4, Contig_13009 as RPM1, Contig_86420 as SGT1 and Contig_29205 as HSP90). In addition, we compared these candidate genes involved in the plant-pathogen interaction pathway with those in *O*. *sativa* (S1 Fig). The results revealed high similarity (at least 95% of similarity) with great majority of these candidate genes except the RPM1-interacting protein (75% of similarity) (S3 Table).

### Genes related to bacterial blight resistance

It was known that wild rice *O*. *meyeriana* is immune to bacterial blight[22]. Therefore, we also identified the contigs involved in the bacterial blight resistance from *O*. *meyeriana* transcripts under native condition. So far 7 bacterial blight resistance genes have been cloned (*Xa1*, *xa5*, *xa13*, *Xa21*, *Xa23*, *Xa26* and *Xa27*). There were 2 types of bacterial blight-resistance contigs (*Xa1* and *Xa26*) identified in *O*. *meyeriana* transcriptome (S4 Table). Four contigs annotated as bacterial blight-resistance protein Xa1 performed significant difference (named OmeXa1_a,b,c,d; S2 Fig). While the 6 contigs annotated as Xa26 have a great similarity with each other as well as Os06g0272000 [GenBank: NP_001057363] and are combined into 4 isoforms (named OmeXa26_a,b,c,d; S3 Fig). The results from expression analysis in different tissues showed that all 4 Xa1 contigs were only expressed in root, while 6 Xa26 contigs displayed significantly different expression in the three tissues. Three of Xa26 contigs (Contig_2863, Contig_3630, Contig_4241) have the highest expression level in leaves, two of Xa26 contigs (Contig_3208, Contig_7391) have the highest expression level in stems and one of Xa26 contigs (Contig_2671) was expressed dominantly in roots.

**Fig 6:**
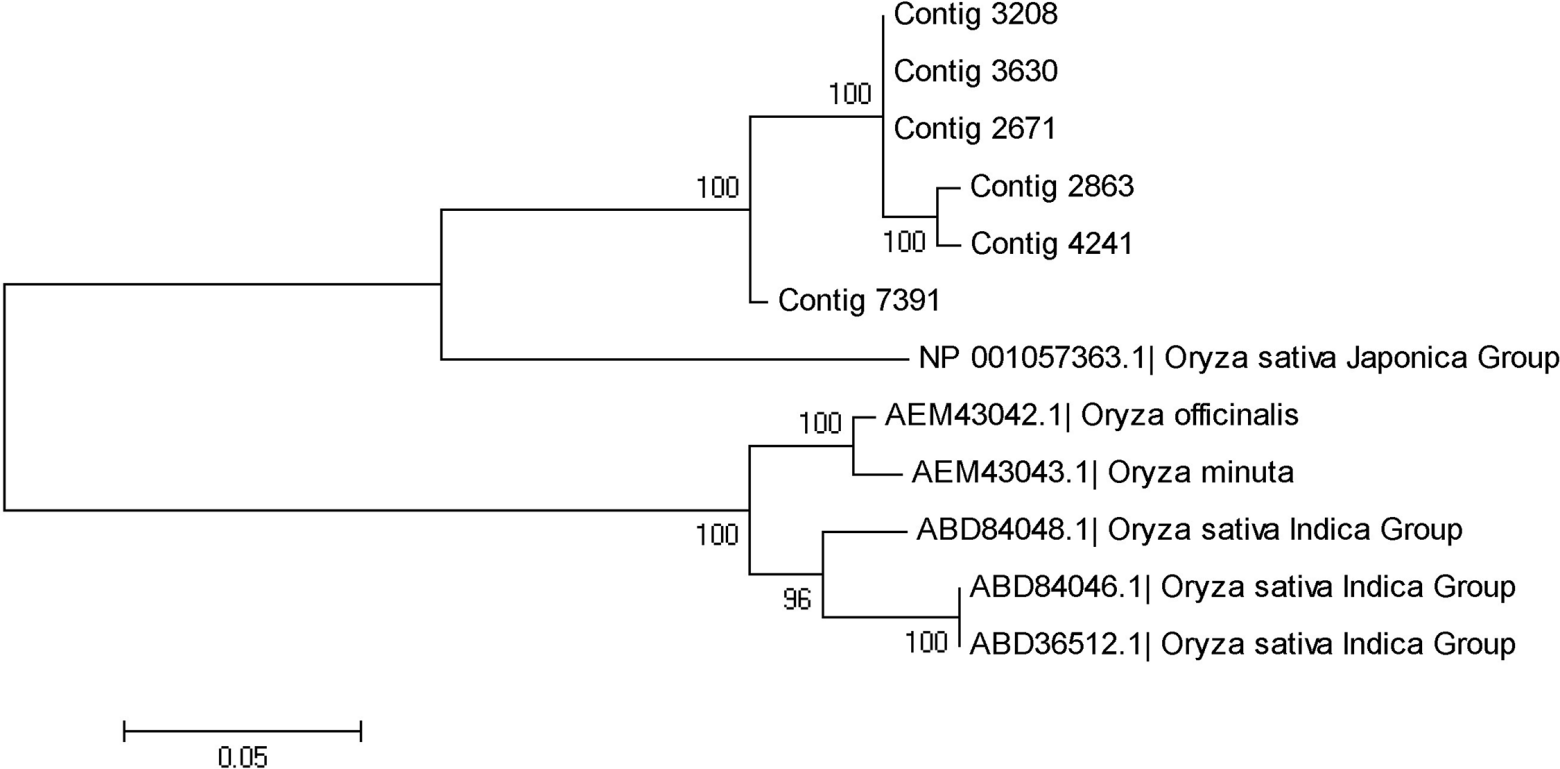
Phylogenetic relationship of the Xa26 proteins of *O*. *meyeriana* with those of other 4 species of *Oryza* genus.

The phylogenetic relationship of OmeX26 was analyzed with other 6 genes from 4 species of *Oryza* genus, including *Oryza sativa Japonica* Group [GenBank: NP_001057363], *Oryza sativa Indica* Group [GenBank: ABD84047, ABD84046, ABD36512], *Oryza officinalis* [GenBank: AEM43042] and *Oryza minuta* [GenBank: AEM43043] by using Neighbor-Joining Method in which the number of Bootstrap is 200 using MEGA 5.05 program. It was clearly shown that the phylogenetic tree is divided into two groups. The Xa26 proteins of *O*. *meyeriana* and *Oryza sativa Japonica* Group were clustered into one branch and those of *Oryza officinalis*, *Oryza minuta* and *Oryza sativa Indica* Group were clustered into another branch (Fig 6). Based on the phylogenetic tree, we also found that the Xa26 protein of *Oryza sativa Indica* Group appeared to be distinct from the sub-branch of *O*. *meyeriana* which grouped closely with *Oryza sativa Japonica* Group and then with *Oryza officinalis*.

### Genes related to other diseases resistance

In addition, among contigs that could not be mapped to the *O*. *sativa* chromosomes, there were 51 contigs annotated as disease resistance protein (S5 Table). These resistance proteins are mainly involved in bacterial speck resistance, rust resistance (including stem rust, stripe rust and leaf rust), fusarium resistance, cyst nematode resistance and downy mildew.

There are 15 contigs annotated as disease resistance proteins containing NBS-LRR (nucleotide biding site-leucine rich repeat) domains. Four of them (Contig_150805, Contig_150973, Contig_151350, Contig_159063) were annotated as NBS-LRR type disease resistance protein rpg1-b, which was related to stem rust resistance. All of the 4 rpg1-b contigs were expressed in leaves or/and stems but had the highest expression level in stems. Six of the rest 11 contigs contained NBS-LRR domains were also expressed in leaves or/and stems and the other five were not identified in stem tissue. In addition, there are 10 contigs (Contig_144680, Contig_145439, Contig_125003, Contig_140592, Contig_149662, Contig_158451, Contig_163238, Contig_165297, Contig_167431, Contig_169234) identified as disease resistance protein rpm1 or rpm1-like, which was involved in bacterial speck resistance. Four of them were expressed in leaf and stem but not in root and the other 6 contigs were specifically expressed in root.

The third most identified disease resistance proteins in *O*. *meyeriana* were cyst nematode resistance proteins. The 9 contigs annotated as cyst nematode resistance protein were all not expressed in the root and two of these (Contig_124975, Contig_166209) were not expressed only in leaves. The details of other disease resistance proteins were shown in S5 Table.

## Discussion

### Transcriptome sequencing of *O*. *meyeriana*

The genus *Oryza* includes two cultivated (2n = 24, AA) and 22 wild species (2n = 24, 48) representing the AA, BB, CC, BBCC, CCDD, EE, FF, GG, KKLL, and HHJJ genome types. Though these wild *Oryza* species are, in fact, grass-like plants which are phenotypically inferior in agronomic traits, such as poor plant type, low grain yield, poor grain type, and are shattering in nature, these wild species are reservoirs of many useful genes, particularly for resistance to major biotic and abiotic stresses[23]. Tremendous progress has been made toward the introgression of important genes from the wild relatives of rice into cultivated rice using classical breeding approaches[24–26]. So far, no introgression could be achieved from *O*. *meyeriana*. Further more, in order to fully utilize and understand the genetic diversity hidden within *O*. *meyeriana*, a more systematic approach must be taken using the tools of transcriptomes.

Next-generation sequencing technology yields a large number of sequences at considerably lower costs compared to traditional sequencing methods, and, therefore, provides a valuable starting point to expedite analysis of less-studied species[12, 27]. Prior to our work, there was no sequencing information on *O*. *meyeriana* in public databases. We used RNA-seq technology for transcriptome profiling to sequence mRNA extracted from leaves, stems and roots. Through this procedure, we obtained a relatively comprehensive transcriptome, which includes 185,323 assembled contigs and 146,450 had annotation information. In addition, we identified 52 contigs as disease resistance protein which could not be mapped to the *O*. *sativa* chromosomes. This study would lay the foundation for finding, identification and usage of new resistance genes and breeding rice resistant varieties.

### KEGG pathways in *O*. *meyeriana*

KEGG is a database resource for understanding high-level functions and utilities of the biological system, such as the cell, the organism and the ecosystem, from molecular-level information, especially large-scale molecular datasets generated by genome sequencing and other high-throughput experimental technologies[28]. It provides a reference knowledge base on the molecular interaction and reaction networks for metabolism, genetic information processing, environmental information processing, cellular processes, organismal systems, human diseases and also on the structure relationships in drug development[29]. In the current study, a total of 8,720 assembled sequences were found to be involved in 300 predicted KEGG metabolic pathways. While in the top 20 pathways with the greatest number of sequences, there were 18 pathways belonged to metabolism pathway. Among the metabolism pathways, carbohydrate metabolism (6) was the most abundant subcategory, including starch and sucrose metabolism, amino sugar and nucleotide sugar metabolism, pyruvate metabolism, glycolysis/gluconeogenesis, pentose and glucuronate interconversions, glyoxylate and dicarboxylate metabolism. Many previous researches have revealed that carbohydrate metabolism is closely related to the plant innate immunity. Fox example, sugar signals contribute to immune responses against pathogens and probably function as priming molecules leading to PTI and ETI in plants[30]. These roles also depend greatly on coordinated relationships with hormones and the light status in an intricate network. Further more, functional approaches have employed to elucidate how alterations in carbohydrate metabolism affect disease development and resistance induction[31]. Expression of the yeast-derived invertase in the cell wall of tobacco and *Arabidopsis* plants leads to the accumulation of carbohydrates and the increased defence gene expression and enhanced resistance against tobacco mosaic virus[32].

### Differences between *O*. *meyeriana* and *O*. *sativa*

The rice genome sequencing has made rice and its wild relatives an increasingly attractive system for biological studies at the genomic level. Considerable insights have been recently gained into understanding the phylogenetic relationships of the genus *Oryza*[33, 34]. Despite extensive studies on evolutionary relationships among rice genomes and species[3, 35], the phylogenetic relationships among rice and its wild relatives remained elusive. In addition, the previous study revealed that *O*. *sativa japonica* rice was first domesticated from a specific population of wild rice, and that *O*. *sativa indica* was subsequently developed from crosses between *japonica* and local wild rice[33]. The puzzles about rice domestication were whether the differentiation between *indica* and *japonica* had occurred in wild rice before domesticated into cultivated rice (*O*. *sativa* L.).

Mapping of sequence reads from *O*. *meyeriana* to *Oryza sativa Japonica* and *Oryza sativa Indica* genomes revealed that there exists small difference on the alignment result from *japonica* (57.09%) and *indica* (57.65%). These results were supported by the comparative analysis with *O*. *sativa* genomes. There were 161467 (87.13%) and 162262 (87.56%) contigs anchored in *japonica* and *indica* genome, respectively, indicating that low divergence existed between *O*. *meyeriana* contigs and *Japonica* or *Indica* genomes. The reason of low reads alignment rates (*japonica*, 57.09%; *indica*, 57.65%) may be the existence of abundant single nucleotide polymorphisms (SNPs). Higher anchoring rate in contig mapping may be due to the fact that blast search program allows more mismatches than bowtie2. In addition, these sequences mapped to all the 12 rice chromosomes showed almost equal distribution (Table 4). The distribution corresponds well to the size of the chromosomes, which highlights the close relationship between *O*. *meyeriana* and *O*. *sativa*. Further more, 19,677 contigs could not be mapped to the *O*. *sativa* chromosomes (including *Japonica* and *Indica*) by homology search against genomic sequences, which may represent *O*. *meyeriana*-specific genes. Among these, only 3,572 contigs had functional annotation and the rest 16,105 contigs may represent new genes in *O*. *meyeriana*.

### Genes involved in plant-pathogen interaction in *O*. *meyeriana*

Plants, unlike animals, lack mobile defender cells and a somatic adaptive immune system. Instead, they rely on the innate immunity of each cell and on systemic signals emanating from infection sites[36, 37]. It is now clear that there are, in essence, two branches of the plant immune system. One uses transmembrane pattern recognition receptors (PRRs) that respond to slowly evolving microbial- or pathogen-associated molecular patterns (MAMPs or PAMPs), such as elongation factor. The second acts largely inside the cells, and occurred with localized programmed cell death to arrest pathogen growth, resulting in cultivar-specific disease resistance[38, 39].

In the present study, we identified 13 genes annotated as encoding enzymes involved in plant-pathogen interaction, including CNCGs, CDPK, CaMCML, FLS2, elf18, CEBiP, WRKY, RIN4, RPM1, SGT1, HSP90, PR1 and JAZ. These genes belonged to two branches of the plant immune system, PAMP-triggered immunity and effector-triggered immunity. In fact, there existed remarkable differences in the two branches of immune systems compared with *O*. *sativa* and *Arabidopsis* (S1 and S4 Figs). PRRs recognize a particular domain of a larger MAMP molecule with structural or enzymatic functions that are crucial for a microbe or pathogen. The genes coding for PRRs specific for flagellin (flg22) and bacterial elongation factor Tu (elf18) epitopes have been identified in plants as FLS2 and EFR, respectively[40, 41]. EFR was not found in *O*. *meyeriana* and *O*. *sativa* while FLS2 and EFR were both existed in *Arabidopsis*. In addition, The best candidates for components of convergent signalling pathways in *O*. *sativa* and *Arabidopsis* were known as mitogen-activated protein kinases (MAPKs)[42]. Several MAPKs involved in *O*. *sativa* defense response have been identified, such as MEKK1, MKK1/2 and MKK4/5, while more MAPKs were found involved in *Arabidopsis* defense response, such as MKK3/6. However, we did not identify MAPKs in defense response in *O*. *meyeriana*. The reason might be that these genes were not existed or not expressed in *O*. *meyeriana* under natural condition. In effector-triggered immunity, there were also two models to achieve layered defensive responses. One model is that resistance (R) proteins recognize perturbations of these virulence targets and thus indirectly recognize the effectors, and the other acts through initiating defense-related gene induction under jasmonic acid (JA) or coronatine[43]. We only identified two R proteins in *O*. *meyeriana* transcripts, RIN4 and RPM1, while another two R proteins were found in *O*. *sativa* and *Arabidopsis*, RPS2 and PBS1. In addition, the latter model is choked because of the absence of transcription factor COI1 and MYC2, which were presented in *O*. *sativa*. The results revealed that the plant-pathogen interaction pathway in *O*. *meyeriana* was more similar with *O*. *sativa*. What is more, there may be less genes or some genes expressed only under special condition involved in plant-pathogen interaction in *O*. *meyeriana* compared with *O*. *sativa*.

### The expression model of resistance genes

According to its characters, genes involved in disease resistance response can be divided into two types: resistance (R) genes and defense genes. R genes, which follows the classical gene-for-gene relationship, could specifically recognize the pathogen Avr (avirulence) gene, either by a direct interaction with its protein product or by an interaction with something made by the Avr gene product if it has a catalytic role[44]. Once this recognition has occurred, defense responses are triggered. The difference of R genes from defense genes is that the former are usually constitutive expression [39, 45].

In the present study, the constitutive expression of many R genes was identified in *O*. *meyeriana* because of all samples collected under native condition, including 2 types of bacterial blight-resistance genes as well as the 51 contigs which were related to other disease resistance and could not be mapped to the *O*. *sativa* chromosomes. In addition, the expression profile of some disease resistance genes in three tissues suggested that their expression level was not related to the location of the diseases. For example, the bacterial blight usually appears leaves but all 4 Xa1 isoforms (Xa1_a,b,c,d) were specifically expressed in root and 2 Xa26 isoforms (Xa26_a,d) have highest expression level in stem (S4 Table). Among the 4 contigs annotated as leaf rust resistance gene, one (Contig_131288) was specifically expressed in root and one (Contig_168093) has the highest expression level in stem (S5 Table).

Therefore, in this study, samples collected from roots, stems and leaves without any treatment were used for RNA-Seq analysis to establish an integrated transcriptome of *O*. *meyeriana* and to survey the characteristics and expression level of R genes in *O*. *meyeriana* under native condition. These results will immensely facilitate the useful gene mining from *O*. *meyeriana*, especially those diseases-resistant genes expressing under the native conditions.

## Methods

### Plant material and RNA extraction

*Oryza meyeriana* was planted at natural temperature and light in Yuanjiang, Yunnan Province of China. Samples of roots, stems and leaves were collected and the collection was permitted by Yunnan Academy of Agricultural Sciences. At least three independent biological replicates of each tissue sample were harvested and immediately frozen in liquid nitrogen.

Total RNA was isolated using the Trizol reagent (Invitrogen, USA) according to the manufacturer’s instructions. The RNA samples were treated with 10 units of DNase I (Fermentas, USA) for 30 min at 37°C to remove the genomic DNA. The RNA concentrations were measured with Qubit fluorometer (Invitrogen, USA) and the RNA quality was verified using the Agilent 2100 Bioanalyzer with a minimum RNA integrated number value of 8.

### Illumina sequencing and quality controls

Qualified total RNA extracted from each sample were used for RNA sequencing by Hiseq 2000. The poly (A)+ RNAs were purified using poly-T oligoattached magnetic beads and eluted with Tris-HCl under heating condition. mRNAs was mixed with fragmentation media and then fragmented. Fragmented mRNAs were copied into first strand cDNA using reverse transcriptase and random primers. This is followed by second strand cDNA synthesis using DNA Polymerase I and RNaseH. Double stranded cDNAs were random fragmented using Nebulizer, then repaired and added an adenine base to the 3’ end. 200 bp cDNAs fragment were purified from a gel and used for further templates enrichment by PCR with two primers that anneal to the ends of the adapters to construct a fragmented cDNAs library. Quality control analysis was performed by Agilent 2100 Bioanalyzer.

The validated 200 bp fragments cDNAs library constructed above was submitted to Illumina Hiseq 2000 to perform transcriptome sequencing. Then the Illumina sequencing by-synthesis, image analysis and base-calling procedures were used to obtain paired-end (PE) read sequence information and base-calling quality values. To ensure that the raw data looks good and there are no problems or biases, pair-end raw reads were performed some simple quality control as implemented by fastqc version 0.10.1[46], including per base sequence quality, per sequence quality scores, per base sequence content, per base GC content and so on. The reads with low scores of less than 20 at 3’ end were filtered out.

### *De novo* assembly and performance evaluation

All the assemblies were performed on a server with 24 cores and 256 GB random access memory. In order to obtain optimal assembling results, we merged PE reads from three tissues into a mixed pool and used various programs for *de novo* assembly of the PE sequence reads to generate a non-redundant set of transcripts. Among the various programs available, we validated publicly available program, Oases V0.2.8[47], which have been developed for assembly of short reads using de Bruijn graph algorithm. In addition, we used other publicly available programs, including Edena V3.130110[48], Soapdenovo V2.04[49] and Trinity_release_20131110[50], which have also been developed for *de novo* assembly of short reads, to obtain best assembly results with our data set. Length distribution was analysed by common perl scripts. N50 number, average length, max length and contig number during different length interval were all been calculated.

Until now, no general criteria have been proposed as standards for evaluation of the quality of transcriptome assembly. We used four substantial factors to assess how well the assembled sequences represent the actual transcriptome population: (i) gene coverage, (ii) transcript sequence quality and completeness, (iii) utilization ratio of reads, (ⅳ) completeness and accuracy[19].

The transcriptome gene coverage was judged by comparison with the sequence information available for *O*. *meyeriana* and phylogenetically related species, *O*. *sativa*. There were 44 complete protein-coding genes from *O*. *meyeriana* were all selected and random 523 proteins from *O*. *sativa* in the NCBI database were chosen as reference databases. The megablast and the common perl analysis script were used to analyze the representation.

Transcriptome quality was assessed by the best candidate coding sequence (CDS) for each contig. Potential coding regions within reconstructed transcripts were analyzed with the perl script in the Trinity package. Finally, to evaluate the utilization ratio of reads, all of the PE reads were aligned back to these contigs by using Bowtie2 (v2.0.0-beta5) program[51]. We calculated the completeness with Com = TP/(TP + FN) (TP = true positives, FN = false negatives), where Com is completeness, TP is the sum of all aligned segment length (the overlap aligned regions were only calculated once), FN is the sum of all reference segment length that were not aligned.

### Functional annotation and classification

The assembled contigs that were longer than 200 bp were compared with the sequences in the NCBI non-redundant protein (NR) databases using the BLASTX algorithm with an E value cut-off of 1e-5. The resulting BLAST hits were processed by Blast2GO software to retrieve associated Gene Ontology (GO) terms describing biological processes, molecular functions, and cellular components. By using specific gene identifiers and accession numbers, Blast2GO produces GO annotations as well as corresponding enzyme commission (EC) numbers for sequences with an E-value ≤1e-5. GO classification was achieved using WEGO [http://wego.genomics.org.cn/cgi-bin/wego/index.pl].

KEGG mapping was used to determine the metabolic pathways[29]. The sequences with corresponding ECs obtained from Blast2GO were mapped to the KEGG metabolic pathway database. To further enrich the pathway annotation and to identify the BRITE functional hierarchies, sequences were also submitted to the KEGG Automatic Annotation Server (KAAS), and the single-directional best hit information method was selected. KAAS annotates every submitted sequence with KEGG orthology (KO) identifiers, which represents an orthologous group of genes directly linked to an object in the KEGG pathways and BRITE (binary relationships of biological entities) functional hierarchy. The Pfam database is a large collection of protein families, each represented by multiple sequence alignments and hidden Markov models (HMMs). Functional domains were predicted through aligning to the Pfam database using HMMER program.

### Sequence composition analysis

The GC content (ratio of guanine and cytosine) and codon usage bias of the transcriptome were analyzed using EMBOSS on the Galaxy platform (http://main.g2.bx.psu.edu/). The perl script program MISA (MIcroSAtellite; http://pgrc.ipk-gatersleben.de/misa/) was used for identification of SSRs. The repeats of di-nucleotides repeats more than 6 times, tri-, tetra-, penta- and hexa-nucleotide repeats more than 5 times were considered as search criteria in MISA script.

### Abundance estimation and differential expression analysis

To investigate the expression level of each contig in different tissues, transcript abundance estimates were obtained by running RSEM separately for each sample, which uses an iterative process to fractionally assign reads to each transcript based on the probabilities of the reads being derived from each transcript. The alignment produced a digital expression levels for each contig and then these were normalized by using perl scripts in Trinity package so as to get FPKM values.

To study expression patterns of transcripts across samples, it is often useful to restrict analysis to those transcripts that are significantly differentially expressed in at least one pairwise sample comparison. Differential expression analysis of the three tissues was performed rely on tools from the Bioconductor project for identifying differentially expressed transcripts, including edgeR[52] and DESeq[53]. Given a set of differentially expressed transcripts, extract those transcripts with FDR≤0.0001 and log_2_fold-change (log_2_FC)≥1.

### Comparison with *Oryza sativa* genome

To investigate the similarity between *Oryza meyeriana* and *Oryza sativa*, we mapped all the sequence reads from three tissues onto the genomic of two *Oryza sativa* varieties, *japonica*-type Nipponbare and *indica*-type 93–11 (http://rise2.genomics.org.cn/page/rice/download.jsp), by using bowtie2 program. Further more, the contigs were aligned to genomic sequences of two *O*. *sativa* varieties by using the BLASTN program with an E-value cut-off of 1e-5. The *O*. *meyeriana* contigs with no matches were subjected to GO analysis for functional classification.

## Supporting Information

S1 Fig The reference plant-pathogen interaction pathway in *O*. *sativa*. (PNG)

S2 Fig Multiple sequences alignment of the 6 contigs annotated as *Xa1*. (EMF)

S3 Fig Multiple sequences alignment of the 6 contigs annotated as *Xa26*. (EMF)

S4 Fig The reference plant-pathogen interaction pathway in *Arabidopsis*. (PNG)

S1 Table The gene coverage statistics of *O*. *meyeriana* transcripts. (XLSX)

S2 Table The codon usage of *O*. *meyeriana* transcripts. (XLSX)

S3 Table The comparison of genes involved in plant-pathogen interaction with *O*. *sativa*. (XLSX)

S4 Table The bacterial blight resistance proteins identified in *O*. *meyeriana*. (XLSX)

S5 Table The disease resistance gene specifically expressed in *O*. *meyeriana*. (XLSX)
