# Supplementary figures and images for: Transcriptomic Analysis and the Expression of Disease-Resistant Genes in Oryza meyeriana under Native Condition
Source: PLoS One. 2015 Dec 7;10(12):e0144518. doi: 10.1371/journal.pone.0144518 (PMC4671656; doi:10.1371/journal.pone.0144518)

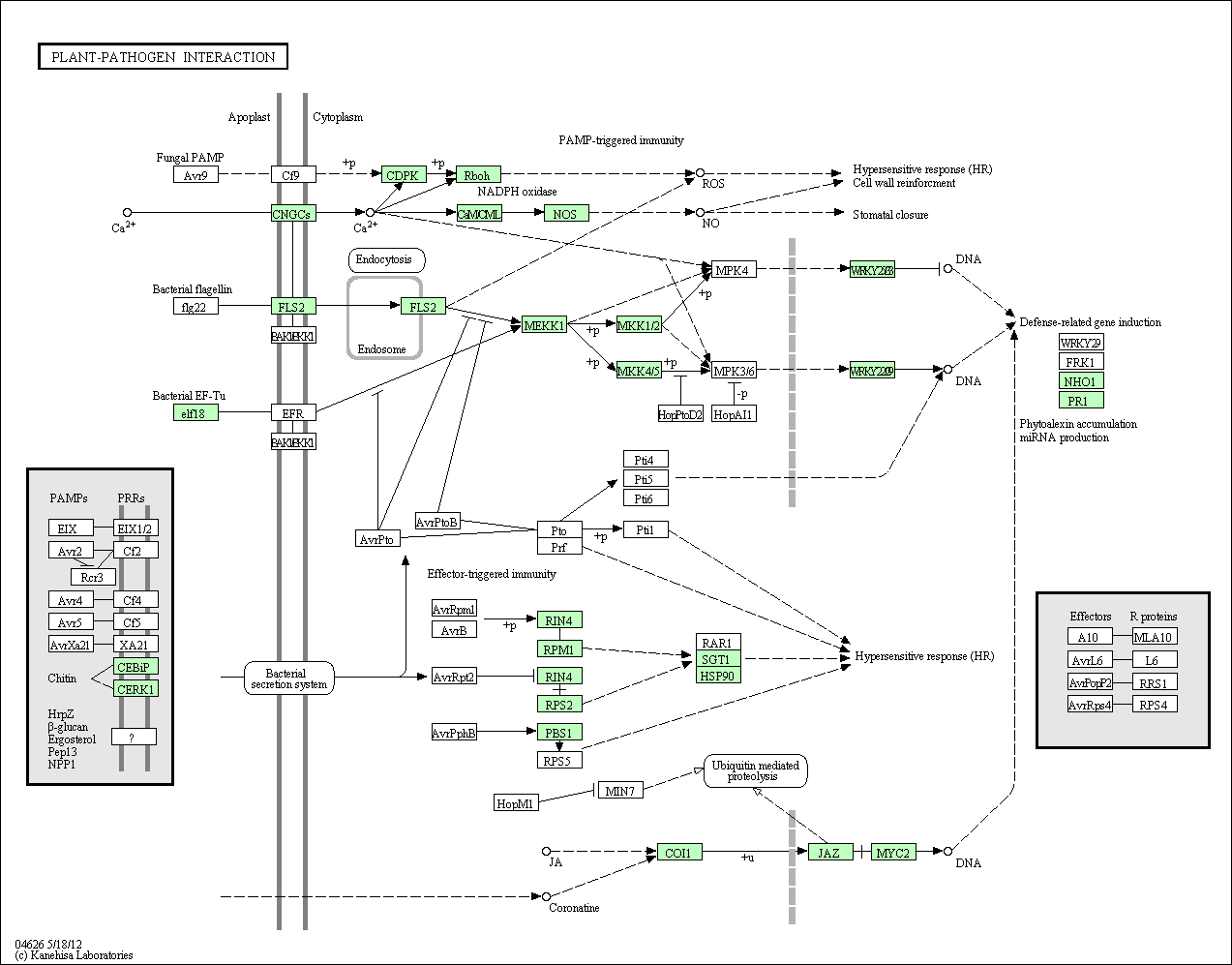

Supplement: S1 Fig — (PNG) [file pone.0144518.s001.png]

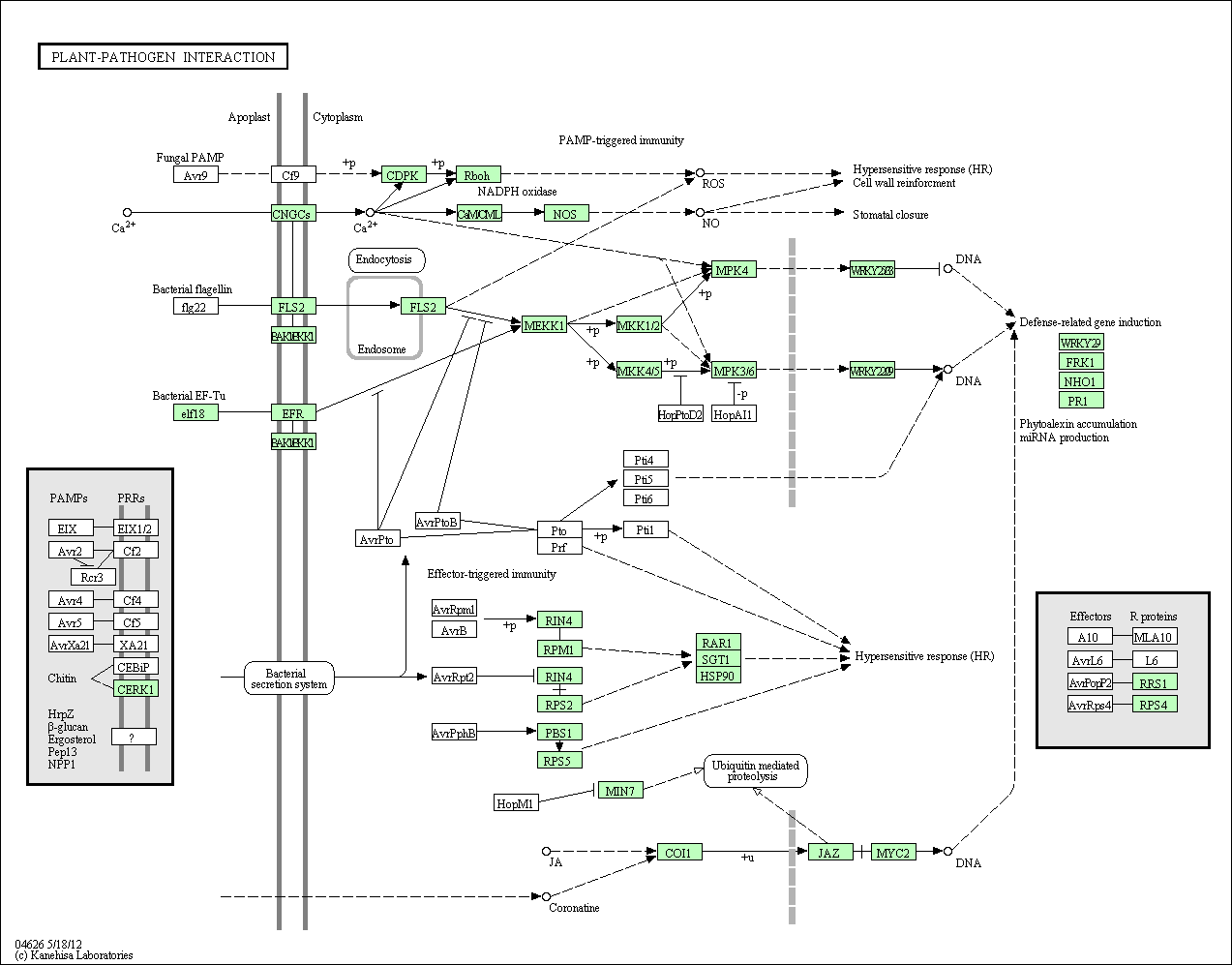

Supplement: S4 Fig — (PNG) [file pone.0144518.s004.png]
